# Supplementary material for: Lipidomic and metabolomic profiles of Coffea canephora L. beans cultivated in Southwestern Nigeria
Source: PLoS One. 2021 Feb 17;16(2):e0234758. doi: 10.1371/journal.pone.0234758 (PMC7888636; doi:10.1371/journal.pone.0234758)
Supplement: S1 Fig — (PDF) [file pone.0234758.s001.pdf]

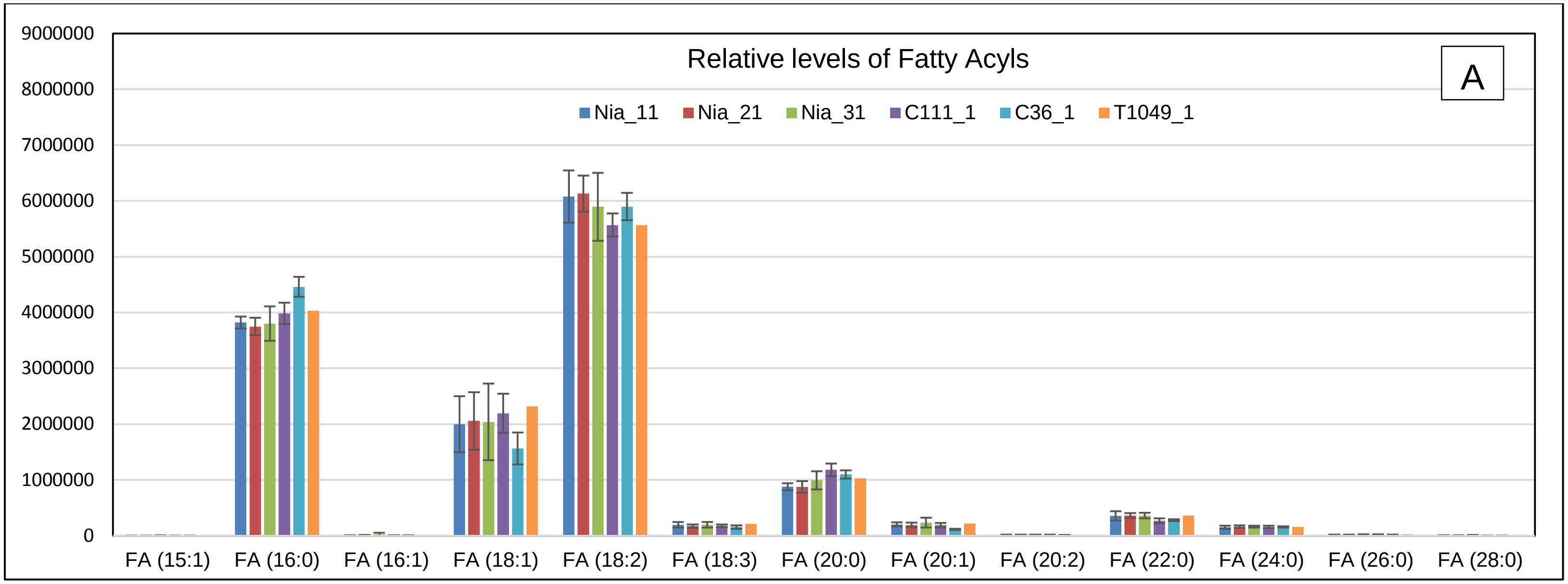
Relative levels of Fatty Acyls
A
Nia_11
Nia_21
Nia_31
C111_1
C36_1
T1049_1
9000000
8000000
7000000
6000000
5000000
4000000
3000000
2000000
1000000
0
FA (15:1)
FA (16:0)
FA (16:1)
FA (18:1)
FA (18:2)
FA (18:3)
FA (20:0)
FA (20:1)
FA (20:2)
FA (22:0)
FA (24:0)
FA (26:0)
FA (28:0)

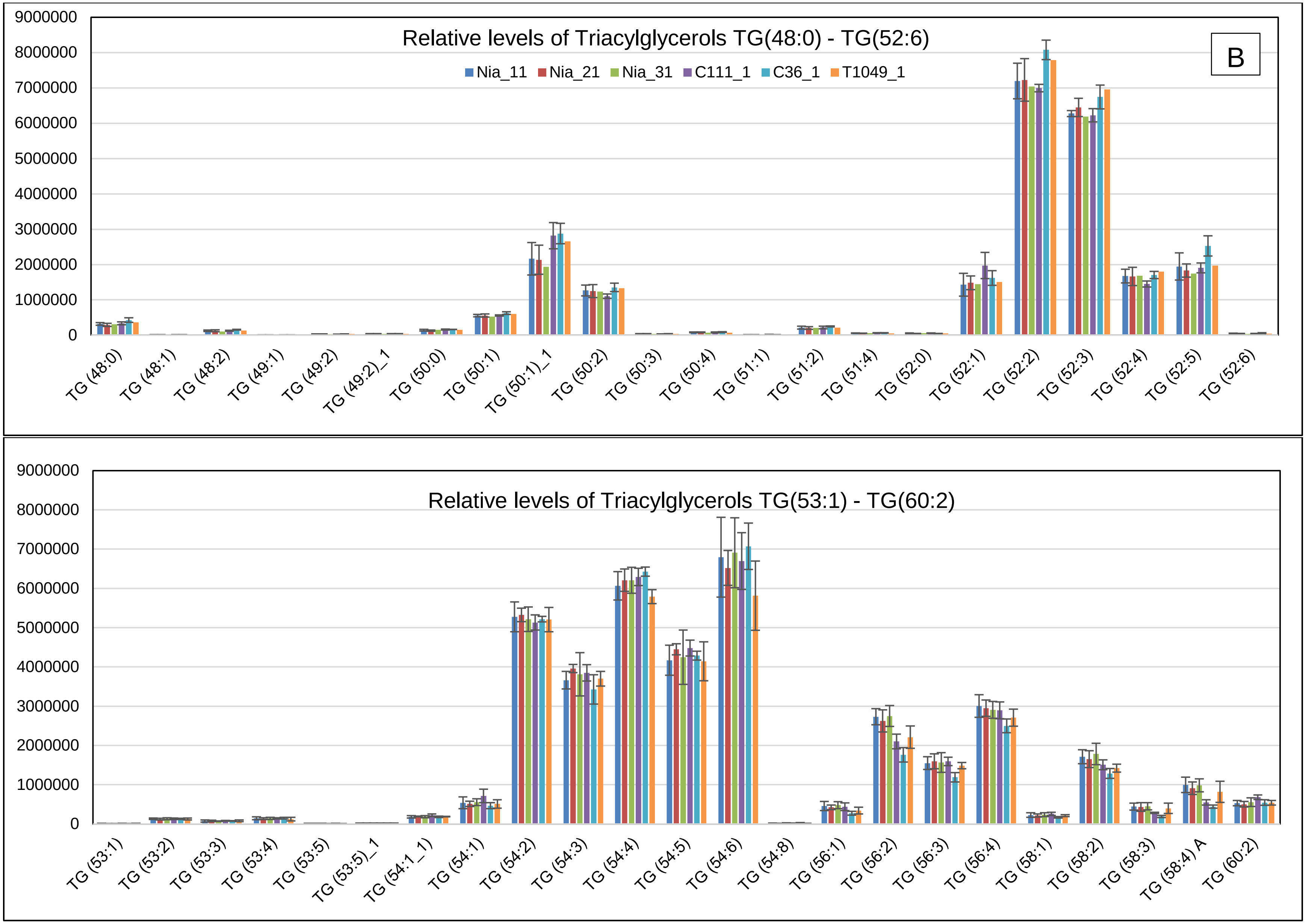
Relative levels of Triacylglycerols TG(48:0) - TG(52:6)
B
Nia_11 Nia_21 Nia_31 C111_1 C36_1 T1049_1
9000000
8000000
7000000
6000000
5000000
4000000
3000000
2000000
1000000
0
TG (48:0) TG (48:1) TG (48:2) TG (49:1) TG (49:2) TG (49:2)_1 TG (50:0) TG (50:1) TG (50:1)_1 TG (50:2) TG (50:3) TG (50:4) TG (51:1) TG (51:2) TG (51:4) TG (52:0) TG (52:1) TG (52:2) TG (52:3) TG (52:4) TG (52:5) TG (52:6)
Relative levels of Triacylglycerols TG(53:1) - TG(60:2)
TG (53:1) TG (53:2) TG (53:3) TG (53:4) TG (53:5) TG (53:5)_1 TG (54:1_1) TG (54:1) TG (54:2) TG (54:3) TG (54:4) TG (54:5) TG (54:6) TG (54:8) TG (56:1) TG (56:2) TG (56:3) TG (56:4) TG (58:1) TG (58:2) TG (58:3) TG (58:4) A TG (60:2)

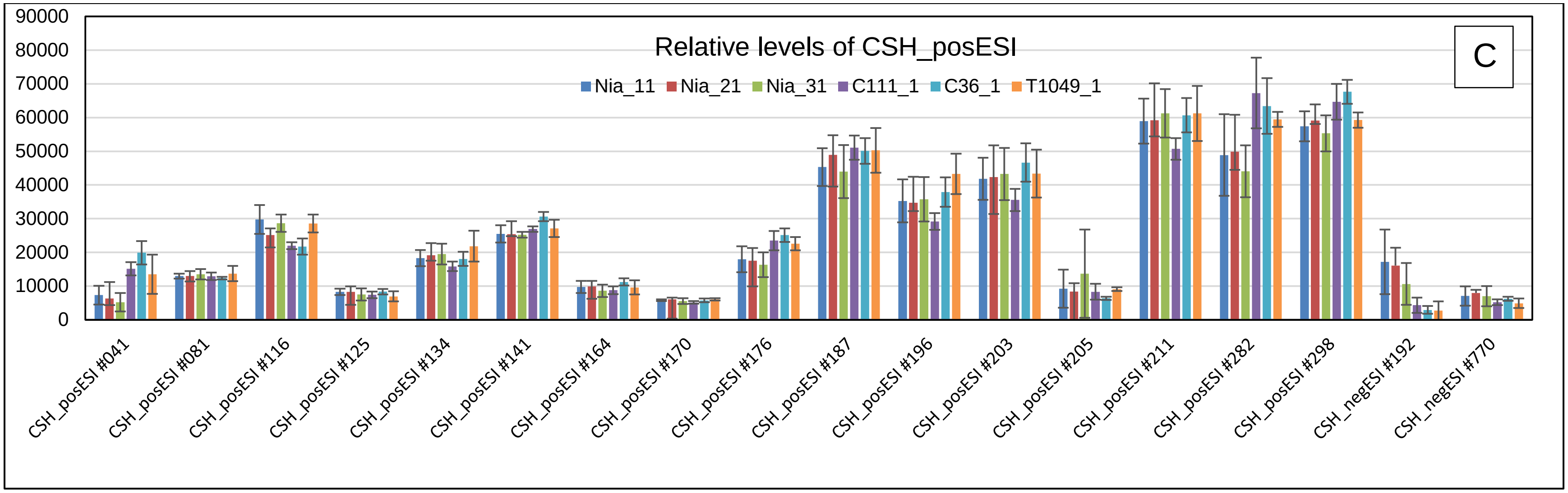
Relative levels of CSH_posESI
C
Nia_11
Nia_21
Nia_31
C111_1
C36_1
T1049_1
90000
80000
70000
60000
50000
40000
30000
20000
10000
0
CSH_posESI #041
CSH_posESI #081
CSH_posESI #116
CSH_posESI #125
CSH_posESI #134
CSH_posESI #141
CSH_posESI #164
CSH_posESI #170
CSH_posESI #176
CSH_posESI #187
CSH_posESI #196
CSH_posESI #203
CSH_posESI #205
CSH_posESI #211
CSH_posESI #282
CSH_posESI #298
CSH_negESI #192
CSH_negESI #770

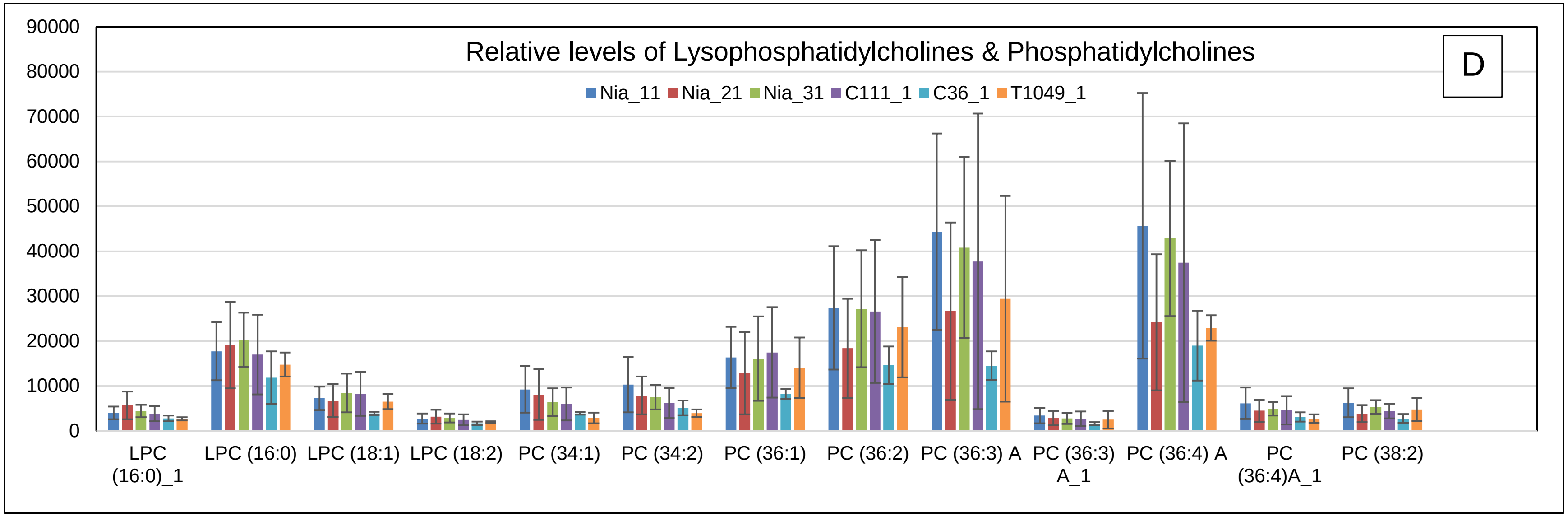

Relative levels of Lysophosphatidylcholines & Phosphatidylcholines
D
Nia_11 Nia_21 Nia_31 C111_1 C36_1 T1049_1
90000
80000
70000
60000
50000
40000
30000
20000
10000
0
LPC (16:0)_1
LPC (16:0)
LPC (18:1)
LPC (18:2)
PC (34:1)
PC (34:2)
PC (36:1)
PC (36:2)
PC (36:3) A
PC (36:3) A_1
PC (36:4) A
PC (36:4)A_1
PC (38:2)

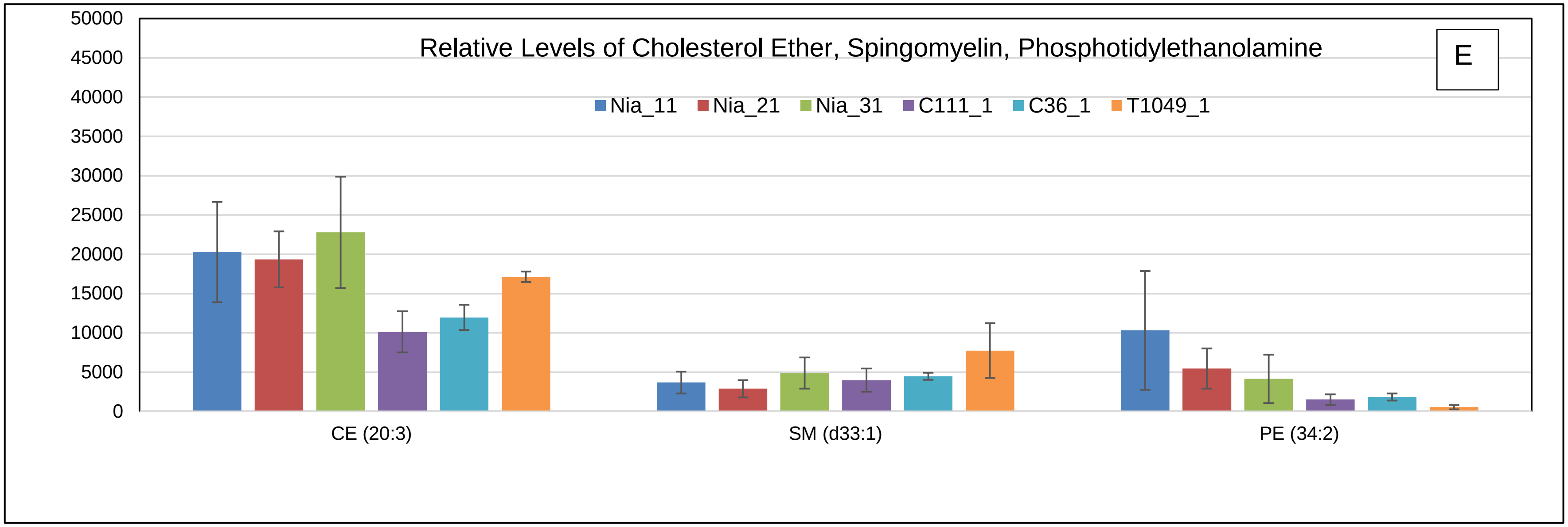
Relative Levels of Cholesterol Ether, Spingomyelin, Phosphotidylethanolamine
E
Nia_11
Nia_21
Nia_31
C111_1
C36_1
T1049_1
50000
45000
40000
35000
30000
25000
20000
15000
10000
5000
0
CE (20:3)
SM (d33:1)
PE (34:2)

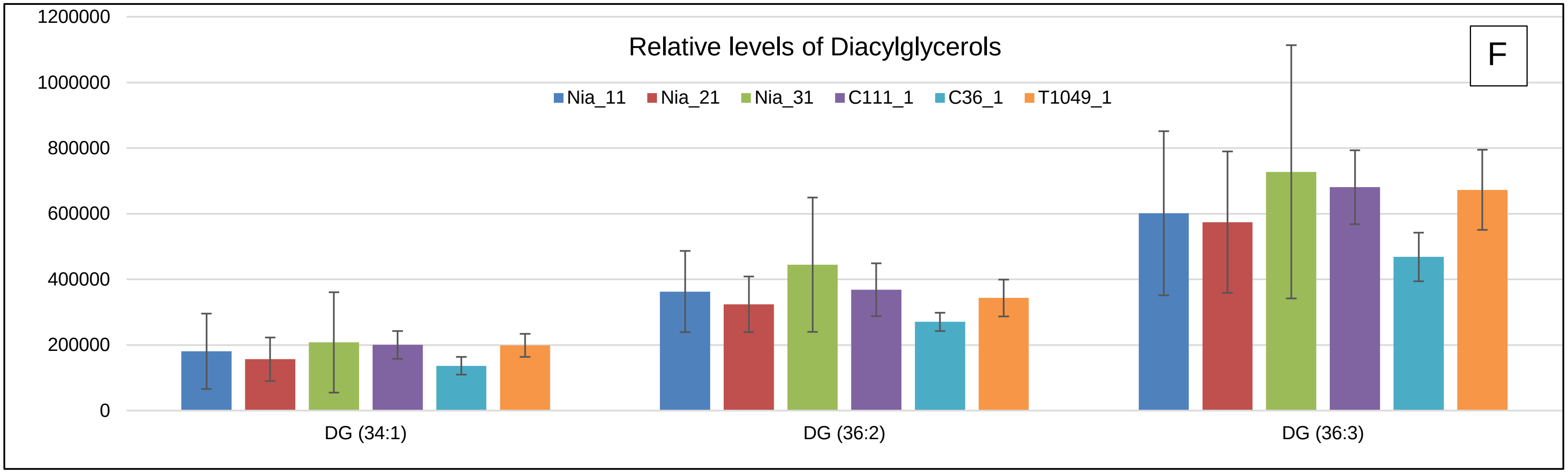

S1 Fig. Bar charts illustrating the relative levels of groups of the different groups of lipids in the *C.canephora* coffees studied. **A**) fatty acyl group, **B**) triacylglycerols **C**) CSH_posESI, **D**) Lysophosphatidylcholine & Phosphatidylcholine, **E**) Diacylglycerol and **F**) Cholesterol Ether, Spingomyelin, and Phosphotidylethanolamine
