# Supplementary material for: Lipidomic and metabolomic profiles of Coffea canephora L. beans cultivated in Southwestern Nigeria
Source: PLoS One. 2021 Feb 17;16(2):e0234758. doi: 10.1371/journal.pone.0234758 (PMC7888636; doi:10.1371/journal.pone.0234758)
Supplement: S1 Table — (PDF) [file pone.0234758.s002.pdf]

S1 Table. The lipid species identified with corresponding charge mode

Positive Lipids	Charge Mode	Negative Lipids	Charge Mode
CE (20:3)	[M+Na]+ _[M+NH4]+	FA (15:1)	[M-H]-
DG (34:1)	[M+Na]+ _[M+NH4]+	FA (16:0)	[M-H]-
DG (36:2)	[M+Na]+ _[M+NH4]+	FA (16:1)	[M-H]-
DG (36:3)	[M+H]+ _[M+Na]+ _[M+NH4]+	FA (18:1)	[M-H]-
LPC (16:0)	[M+H]+	FA (18:2)	[M-H]-
LPC (18:1)	[M+H]+	FA (18:3)	[M-H]-
PC (36:1)	[M+H]+	FA (20:0)	[M-H]-
PC (36:2)	[M+H]+	FA (20:1)	[M-H]-
PC (36:3) A	[M+H]+	FA (20:2)	[M-H]-
PC (36:4) A	[M+H]+	FA (22:0)	[M-H]-
PC (38:2)	[M+H]+	FA (24:0)	[M-H]-
SM (d33:1)	[M+H]+	FA (26:0)	[M-H]-
TG (48:0)	[M+Na]+ _[M+NH4]+	FA (28:0)	[M-H]-
TG (48:1)	[M+NH4]+	LPC (16:0)	[M+HAc-H]-
TG (48:2)	[M+Na]+ _[M+NH4]+	LPC (18:2)	[M+HAc-H]-
TG (49:1)	[M+NH4]+	PC (34:1)	[M+HAc-H]-
TG (49:2)	[M+Na]+	PC (34:2)	[M+HAc-H]-
TG (49:2)	[M+NH4]+	PC (36:3) A	[M+HAc-H]-
TG (50:0)	[M+Na]+ _[M+NH4]+	PC (36:4) A	[M+HAc-H]-
TG (50:1)	[M+Na]+	PE (34:2)	[M-H]-
TG (50:1)	[M+NH4]+	CSH_negESI #192	[M-H]-
TG (50:2)	[M+Na]+	CSH_negESI #770	[M-H]-
TG (50:3)	[M+Na]+		
TG (50:4)	[M+Na]+ _[M+NH4]+		
TG (51:1)	[M+NH4]+		
TG (51:2)	[M+Na]+ _[M+NH4]+		
TG (51:4)	[M+Na]+ _[M+NH4]+		
TG (52:0)	[M+NH4]+		
TG (52:1)	[M+Na]+ _[M+NH4]+		
TG (52:2)	[M+Na]+ _[M+NH4]+		
TG (52:3)	[M+Na]+ _[M+NH4]+		
TG (52:4)	[M+Na]+		
TG (52:5)	[M+H]+ _[M+Na]+ _[M+NH4]+		
TG (52:6)	[M+Na]+ _[M+NH4]+		
TG (53:1)	[M+Na]+ _[M+NH4]+		
TG (53:2)	[M+Na]+ _[M+NH4]+		
TG (53:3)	[M+Na]+ _[M+NH4]+		

TG (53:4)	[M+Na]+ _[M+NH4]+		
TG (53:5)	[M+Na]+		
TG (53:5)	[M+Na]+ _[M+NH4]+		
TG (54:1)	[M+Na]+		
TG (54:1)	[M+NH4]+		
TG (54:2)	[M+Na]+ _[M+NH4]+		
TG (54:3)	[M+Na]+ _[M+NH4]+		
TG (54:4)	[M+H]+ _[M+Na]+ _[M+NH4]+		
TG (54:5)	[M+Na]+ _[M+NH4]+		
TG (54:6)	[M+H]+ _[M+Na]+ _[M+NH4]+		
TG (54:8)	[M+NH4]+		
TG (56:1)	[M+Na]+ _[M+NH4]+		
TG (56:2)	[M+Na]+ _[M+NH4]+		
TG (56:3)	[M+Na]+ _[M+NH4]+		
TG (56:4)	[M+Na]+ _[M+NH4]+		
TG (58:1)	[M+Na]+ _[M+NH4]+		
TG (58:2)	[M+Na]+ _[M+NH4]+		
TG (58:3)	[M+Na]+ _[M+NH4]+		
TG (58:4) A	[M+Na]+ _[M+NH4]+		
TG (60:2)	[M+Na]+ _[M+NH4]+		
CSH_posESI #022	[M+H]+		
CSH_posESI #041	[M+H]+		
CSH_posESI #081	[M+H]+		
CSH_posESI #116	[M+H]+		
CSH_posESI #125	[M+H]+		
CSH_posESI #134	[M+H]+		
CSH_posESI #141	[M+H]+		
CSH_posESI #164	[M+H]+		
CSH_posESI #170	[M+H]+		
CSH_posESI #176	[M+H]+		
CSH_posESI #187	[M+H]+		
CSH_posESI #196	[M+H]+		
CSH_posESI #203	[M+H]+		
CSH_posESI #205	[M+H]+		
CSH_posESI #211	[M+H]+		
CSH_posESI #282	[M+H]+		
CSH_posESI #298	[M+H]+		
