# Supplementary material for: Lipidomic and metabolomic profiles of Coffea canephora L. beans cultivated in Southwestern Nigeria
Source: PLoS One. 2021 Feb 17;16(2):e0234758. doi: 10.1371/journal.pone.0234758 (PMC7888636; doi:10.1371/journal.pone.0234758)
Supplement: S2 Table — (PDF) [file pone.0234758.s003.pdf]

S2 Table. Pearson's Correlation Coefficient Values, *P*-values and Adjusted *P*-values for 20 Lipid-to-Lipid Associations. The strongest positive and negative correlations are shown. All analyses can be found in Data File 1

Metabolite 1	Metabolite 2	Correlation Coefficient	*p*-value	Adjusted -*p*-values
**Positive Correlations**				
TG..50.1._1	CSH_posESI..282	0.9712413	2.48E-15	1.25E-11
CSH_posESI..203	CSH_posESI..211	0.96956129	2.72E-15	1.25E-11
DG..34.1.	DG..36.3.	0.9628317	2.85E-14	8.75E-11
TG..50.1._1	CSH_posESI..176	0.96014177	5.59E-14	1.29E-10
TG..50.2.	CSH_posESI..203	0.95785921	9.61E-14	1.77E-10
PC..34.2.	PC..36.4.A_1	0.95630251	1.32E-13	2.02E-10
LPC..16.0._1	LPC..18.2.	0.94164473	5.92E-12	7.79E-09
TG..50.2.	CSH_posESI..211	0.93720174	1.41E-11	1.62E-08
PC..36.1.	PC..36.2.	0.91832211	4.40E-10	4.5105E-07
TG..50.1._1	CSH_posESI..282	0.9712413	2.48E-15	1.25E-11
**Negative Correlations**				
CE..20.3.	CSH_posESI..298	-0.8806894	2.42E-08	8.5717E-06
CE..20.3.	CSH_posESI..282	-0.8529894	2.6432E-07	6.5838E-05
CE..20.3.	TG..50.1._1	-0.838215	7.3804E-07	0.00014786
CE..20.3.	CSH_posESI..176	-0.8094119	4.5655E-06	0.00068976
TG..58.3.	CSH_posESI..141	-0.8042666	6.1361E-06	0.00086832
TG..58.3.	FA..16.0.	-0.8041921	6.1361E-06	0.00086832
CSH_posESI..164	FA..20.2.	-0.783955	1.563E-05	0.00165569
TG..50.2.	TG..54.1.	-0.7801918	1.903E-05	0.00196486
CE..20.3.	TG..52.1.	-0.7801385	1.903E-05	0.00196486
TG..54.1.	CSH_posESI..203	-0.7740364	2.409E-05	0.00217642
